# A shallow convolutional neural network for blind image sharpness assessment

**DOI:** 10.1371/journal.pone.0176632

**Published:** 2017-05-01

**Authors:** Shaode Yu, Shibin Wu, Lei Wang, Fan Jiang, Yaoqin Xie, Leida Li

**Affiliations:** 1 Shenzhen Institute of Advanced Technology, Chinese Academy of Sciences, Shenzhen, Guangdong, China; 2 Shenzhen College of Advanced Technology, University of Chinese Academy of Sciences, Shenzhen, Guangdong, China; 3 Faculty of Information Engineering and Automation, Kunming University of Science and Technology, Kunming, Yunnan, China; 4 School of Information and Control Engineering, China University of Mining and Technology, Xuzhou, Jiangsu, China; Huazhong University of Science and Technology, CHINA

## Abstract

Blind image quality assessment can be modeled as feature extraction followed by score prediction. It necessitates considerable expertise and efforts to handcraft features for optimal representation of perceptual image quality. This paper addresses blind image sharpness assessment by using a shallow convolutional neural network (CNN). The network takes single feature layer to unearth intrinsic features for image sharpness representation and utilizes multilayer perceptron (MLP) to rate image quality. Different from traditional methods, CNN integrates feature extraction and score prediction into an optimization procedure and retrieves features automatically from raw images. Moreover, its prediction performance can be enhanced by replacing MLP with general regression neural network (GRNN) and support vector regression (SVR). Experiments on Gaussian blur images from LIVE-II, CSIQ, TID2008 and TID2013 demonstrate that CNN features with SVR achieves the best overall performance, indicating high correlation with human subjective judgment.

## Introduction

A picture wins a thousand words. With the rapid pace of modern life and the massive dissemination of smart phones, digital images have been a major source of information acquisition and distribution. Since an image is prone to various kinds of distortions from its capture to the final display on digital devices, a lot of attention has been paid to the assessment of perceptual image quality [1–8].

Subjective image quality assessment (IQA) is the most straightforward. However, it is laborious and may introduce bias and errors. Comparatively, objective evaluation of visual image quality with full- or reduced-reference based methods enables impartial judgment [9–22]. These algorithms have reached high-level performance, while in most possible situations, the reference messages are not easy or impossible to acquire. Thus, no-reference or blind IQA methods are more useful in real applications [23–34].

Blind image quality assessment (BIQA) mainly consists of two steps, feature extraction (*T*) and score prediction (*f*). Before rating an image, *T* and *f* should be prepared. The former aims to select optimal features for image quality representation, while the latter builds the functional relationship between the features and subjective scores. With considerable expertise and efforts, a BIQA system can be built. As such, a test image (*I*) is input to the system and represented with features (*T*). Finally, the function *f* will quantify the features and figure out a numerical score (*s*) as the output, denoting the predicted quality of the test image. The procedure for score prediction can be formulated as follows,

s=f(T(I)). (1)

Blind image sharpness assessment (BISA) is studied in this paper. Among various kinds of distortions, sharpness is commonly degraded by camera out-of-focus, relative target motion and lossy image compression. It is crucial to readability and content understanding. Sharpness is inversely related to blur which is typically determined by the spread of edges in the spatial domain, and accordingly the attenuation of high frequency components. Karam *et* *al*. [35] introduced the Just Noticeable Blur (JNB) model and integrated local contrast and edge width in each edge blocks into a probability summation model. Later, they improved the model with the cumulative probability of blur detection (CPBD) [36]. Ciancia *et* *al*. [37] selected blur-related features as the input of a neural network and realized no-reference blur assessment with multi-feature classifiers. Vu *et* *al*. [38] combined two features, the high frequency content with the slope of local magnitude spectrum and the local contrast with total variation, to form the spectral and spatial sharpness (S3) index. Vu *et* *al*. [39] defined a fast image sharpness (FISH) metric which weights the log-energies of wavelet coefficients. Hassen *et* *al*. [40] explored the strength of local phase coherence (LPC) based on the observation that blur disrupts image LPC structures. Sang *et* *al*. [41, 42] used the shape of singular value curve (SVC) to measure the extent of blur, because the extent of blur results in attenuation of singular values. Bahrami and Kot [43] took account of maximum local variation (MLV) of each pixel and utilized the standard deviation of ranking weighted MLVs as the sharpness score. Li *et* *al*. [44] proposed the sparse representation based image sharpness (SPARISH) model that utilizes dictionary learning of natural image patches. Gu *et* *al*. [45] designed an autoregressive based image sharpness metric (ARISM) via image analysis in the autoregressive parameter space. Li *et* *al*. [46] presented a blind image blur evaluation (BIBLE) index which characterizes blur with discrete moments, because noticeable blur affects the moment magnitudes of images.

Deep learning has revolutionized image representation and shed light on utilizing high-level features for BIQA [47, 48]. Li *et* *al*. [49] adapted Shearlet transform for spatial feature extraction and employed a deep network for image score regression. Hou and Gao [50] recast BIQA as a classification problem and used a saliency-guided deep framework for feature retrieval. Li *et* *al*. [51] took the Prewitt magnitudes of segmented images as the input of convolutional neural network (CNN). Lv *et* *al*. [52] explored the local normalized multi-scale difference of Gaussian response as features and designed a deep network for image quality rating. Hou *et* *al*. [53] designed a deep learning model trained by deep belief net and then fine-tuned it for image quality estimation. Yet it is found that some deep learning based methods need to handcraft features [49–52] or redundant operations [50, 52, 53].

This paper presents a shallow CNN to address BISA. On the one hand, several studies indicate that image sharpness is generally characterized by the spread of edge structures [35–38, 44, 46]. Interestingly, what CNN learns in the first layer are mainly edges [47, 48]. Thus, it is intuitive to design a single feature layer CNN for image sharpness estimation. On the other hand, small data sets make deep networks hard to converge which may increase the risk of over-fitting. Consequently, a shallow CNN can be well trained with limited samples [54]. To the best of our knowledge, the most similar work is Kang’s CNN [55]. The network utilizes two full-connection layers and obtains dense features by both maximum and minimum pooling before image scoring. Relatively, our network is much simpler in the architecture and more suitable for the analysis of small databases. Besides, our CNN is verified with Gaussian blurring images from four popular databases. After features are retrieved for representation of sharpness, the prediction performance of multilayer perceptron (MLP) is compared to both general regression neural network (GRNN) [56] and support vector regression (SVR) [57]. In the end, the effect of color information on our CNN and the running time are reported.

## A shallow CNN

**Fig 1:**
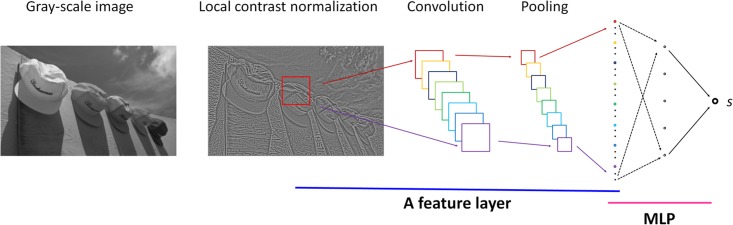
The proposed BISA system. A gray-scale image is pre-processed with local contrast normalization and then a number of image patches are randomly cropped for CNN training, validation and final testing.

The simplified CNN consists of one feature layer and the feature layer is made up of convolutional filtering and average pooling. As shown in Fig 1, a gray-scale image is pre-processed with local contrast normalization. Then, a number of image patches are randomly cropped for feature extraction. At last, the features are as input to MLP for score prediction. By supervised learning, parameters in the network are updated and fine-tuned with back-propagation.

### Feature extraction

#### Local contrast normalization

It has a decorrelating effect in spatial image analysis by applying a local non-linear operation to remove local mean displacements and to normalize the local variance [25, 58]. As in [52, 55], the local normalization is formulated as following,

$$\tilde{I}\left(i,j\right)=\frac{I(i,j)-\mu (i,j)}{\sigma (i,j)+C},$$ (2)

where,

$$\mu \left(i,j\right)=\frac{1}{\left(2P+1)(2Q+1\right)}\sum_{p=-P}^{p=P}\sum_{q=-Q}^{q=Q}I\left(i+p,j+q\right),$$ (3)

and

$$\sigma^{2}\left(i,j\right)=\sum_{p=-P}^{p=P}\sum_{q=-Q}^{q=Q}{\left(I\left(i+p,j+q\right)-\mu \left(i,j\right)\right)}^{2}.$$ (4)

In the equations, *I*(*i*, *j*) is the pixel intensity value at (*i*, *j*), $\tilde{I}\left(i,j\right)$ is its normalized value, *μ*(*i*, *j*) is the mean value, *σ*(*i*, *j*) is the standard deviation and *C* is a positive constant (*C* = 10). Besides, [2*P* + 1, 2*Q* + 1] is the window size and *P* = *Q* = 3.

#### Feature representation

Each patch randomly cropped in the pre-processed image is through convolutional filtering and pooling before full connection to MLP. A feature vector of an image patch is generated and formulated as,

$$X=T\left(I_{p}\right)={\left(x_{1},\ldots ,x_{l},\ldots ,x_{n}\right)}^{{}^{\prime }},$$ (5)

where *I*_*p*_ is an image patch, *n* is the feature dimension and *x*_*l*_ is the *l*^*th*^ component of the feature vector *X*.

### Score prediction

#### Multilayer perceptron (MLP)

**Fig 2:**
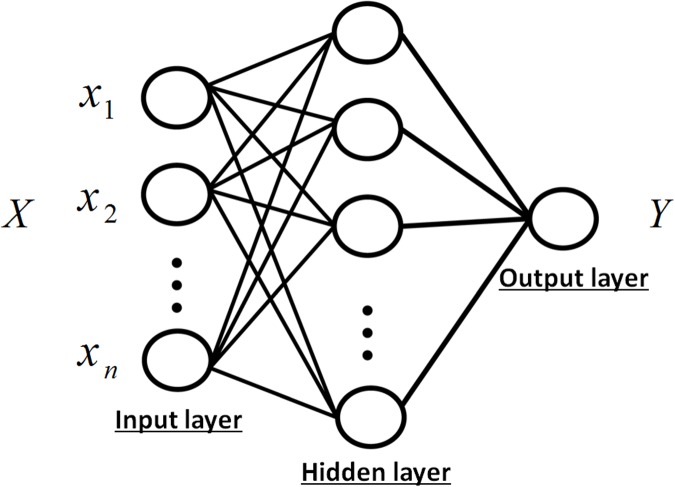
MLP with one hidden layer. It consists of three layers, the input layer, the hidden layer and the output layer.

Fig 2 illustrates an MLP with a hidden layer. The output *f*(*X*) with regard to the input feature *X* can be expressed as following,

$$f\left(X\right)=f_{mlp}\left(w,b;X\right),$$ (6)

where *f*_*mlp*_ denotes an activation function, while **w** and **b** respectively stand for the weight vector and the bias vector.

#### General regression neural network (GRNN)

**Fig 3:**
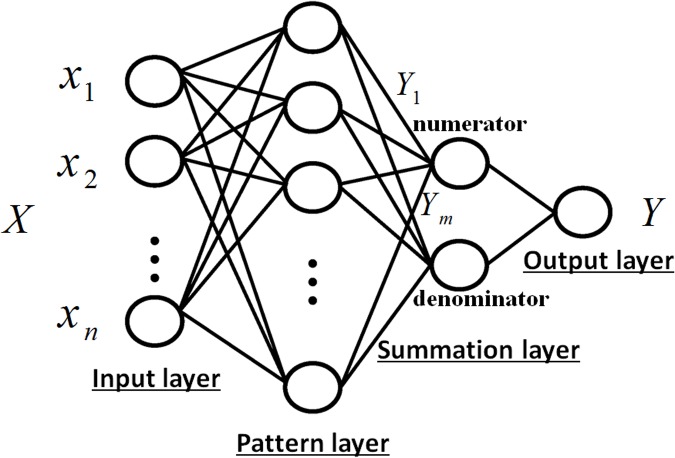
A semantic description of GRNN. It consists of four layers, the input layer, the pattern layer, the summation layer and the output layer.

GRNN is a powerful regression tool based on statistical principles [56]. It takes only a single pass through a set of feature instances and requires no iterative training. GRNN consists of four layers as shown in Fig 3. Assume that *m* samples ${\left\{X_{i},Y_{i}\right\}}_{i=1}^{m}$ have been used to train the GRNN. To an input feature vector *X*, its output *f*(*X*) can be described as below,

$$f\left(X\right)=f_{grnn}\left(X\right)=\frac{\sum_{i=1}^{n}Y_{i}e^{-{(X-X_{i})}^{{}^{\prime }}\left(X-X_{i}\right)/2\sigma^{2}}}{\sum_{i=1}^{n}e^{-{\left(X-X_{i}\right)}^{{}^{\prime }}\left(X-X_{i}\right)/2\sigma^{2}}},$$ (7)

where *Y*_*i*_ is the weight between the *i*^*th*^ neuron in the pattern layer and the numerator neuron in the summation layer, and *σ* is a spread parameter. In GRNN, only *σ* is tunable and a larger value leads to a smoother prediction.

#### Support vector regression (SVR)

SVR is effective in handling numerical prediction in high dimension space [57, 59]. For an input *X*, the goal of *ε*-SVR is to find a function *f*(*X*) that has the maximum deviation of *ε* from the subjective score *Y* for all the training patches. The function is defined by

$$f\left(X\right)=f_{svr}\left(X\right)=w^{{}^{\prime }}\varphi \left(X\right)+\gamma ,$$ (8)

where *φ*(⋅) is a nonlinear function, **w** is a weight vector and *γ* is a bias. The aim is to find **w** and *γ* from the training data such that the error is less than a predefined value of *ε*. The radial basis function is used as the kernel function, *K*(*X*_*i*_, *X*) = *e*^−*ρ*||*X*_*i*_−*X*||^, and *ρ* is a positive parameter that controls the radius and *X*_*i*_ is a training sample. By using a validation set to tradeoff the prediction error, *ρ* and *ε* are determined [60].

### Network training

CNN is end-to-end trained by supervised learning with stochastic gradient descent. Assume there are a set of features ${\left\{X_{i}\right\}}_{i=1}^{n}$ and corresponding scores ${\left\{Y_{i}\right\}}_{i=1}^{n}$. The training aims to minimize the loss function *L*(**w**, **b**),

$$\begin{array}{ccc} L(w,b) & = & \frac{1}{n}\sum_{i=1}^{n}(\frac{1}{2}\left|\right|Y_{i}-s_{i}{\left|\right|}^{2})\\ & = & \frac{1}{n}\sum_{i=1}^{n}(\frac{1}{2}\left|\right|Y_{i}-f_{mlp}\left(w,b;X_{i}\right){\left|\right|}^{2}), \end{array}$$ (9)

which is the sum of square error between the predicted *s*_*i*_ and the subjective score *Y*_*i*_.

Using gradient descent, the relationship between the *l*^*th*^ and the (*l* + 1)^*th*^ iteration to each weight component can be described as following,

$$w^{l+1}=\mu w^{l}-\eta \frac{\partial L(w,b)}{\partial w^{l}},$$ (10)

$$b^{l+1}=\mu b^{l}-\eta \frac{\partial L(w,b)}{\partial b^{l}},$$ (11)

where *μ* is the momentum that indicates the contribution of the previous weight update in the current iteration, and *η* denotes the learning rate.

## Experiments

### Images for performance evaluation

**Fig 4:**
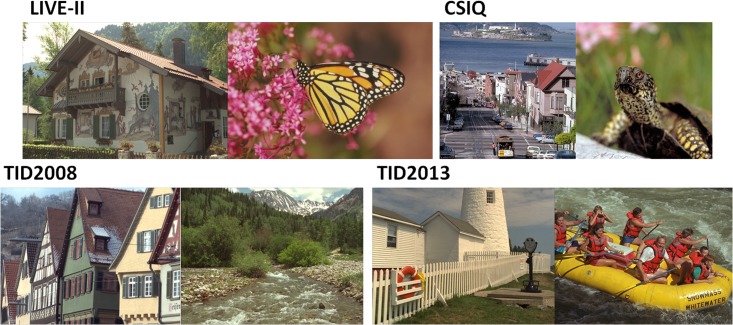
Example of Gaussian blurring images in four databases.

Gaussian blurring images are collected from four popular databases. LIVE-II [10] and CSIQ [61] respectively contain 29 and 30 reference images which are distorted with 5 blur levels and scored by differential mean opinion scores (DMOS). Both TID2008 [62] and TID2013 [63] have 25 references and use mean opinion scores (MOS) for scoring. Each reference image in TID2008 and TID2013 is degraded with 4 and 5 different blur levels, respectively. Fig 4 shows some representative images.

### Experiment design

LIVE-II is taken as the baseline database for tuning parameters in CNN, GRNN and SVR. Blurred images in LIVE-II are portioned into 20:4:5 for training, validation and test, respectively. After that, parameters in GRNN and SVR are optimized based on learned features from CNN. In the end, about 60%, 20% and 20% blurring images in each database are randomly selected for training, validation and test, respectively.

Besides Kang’s CNN [55], ten state-of-the-art BISA methods are evaluated. These methods are JNB [35], CPBD [36], S3 [38], FISH [39], LPC [40], SVC [42], MLV [43], SPARISH [44], ARISM [45] and BIBLE [46]. In the end, the running time of involved algorithms and the effect of color information on our CNN are studied.

### Performance criteria

Two criteria are recommended for IQA performance evaluation by the video quality experts groups (VQEG, http://www.vqeg.org). Pearson linear correlation coefficient (PLCC) evaluates the prediction accuracy, while Spearman rank-order correlation coefficient (SROCC) measures the prediction monotonicity. Values of both criteria range in [0, 1] and higher value indicates better rating prediction.

A nonlinear regression is first applied to map the predicted scores to subjective human ratings using a five-parameter logistic function as follows,

$$Q\left(s\right)=q_{1}\left(\frac{1}{2}-\frac{1}{1+e^{q_{2}\left(s-q_{3}\right)}}\right)+q_{4}s+q_{5},$$ (12)

where *s* and *Q*(*s*) are the input score and the mapped score, and *q*_*i*_ (*i* = 1, 2, 3, 4, 5) are determined during the curve fitting.

### Software and platform

Softwares are run on Linux system (Ubuntu 14.04). The system is embedded with 8 Intel Xeon(R) CPU (3.7GHz), 16GB DDR RAM and one GPU card (Nvidia 1070). Kang’s CNN is implemented by us following the paper [55]. Both CNN models are realized with Theano 0.8.2 (Python 2.7.6) and accessible on GitHub at present for fair comparison (https://github.com/Dakar-share/Plosone-IQA). Other codes are realized with Matlab. Ten BISA methods are provided by authors and estimated without any modifications, GRNN is with the function *newgrnn* and SVR is from LIBSVM [59].

## Result

### Parameter tuning

Several parameters are experimentally determined, the patch number per image (*P*_*n*_), the kernel number (*K*_*n*_) and the kernel size ([*K*_*x*_, *K*_*y*_]) in feature extraction, and the iteration number (*N*_*i*_) in network training. In addition, the spread parameter (*σ*) in GRNN and cost function (*c*) in *ε*-SVR are also studied. Note that in the network, we define the size of image patch [16 16], the learning rate *η* = 0.01, the bias *γ* = 0.1 and the momentum *μ* = 0.9, and other parameters are set by default.

#### Parameters in CNN

**Fig 5:**
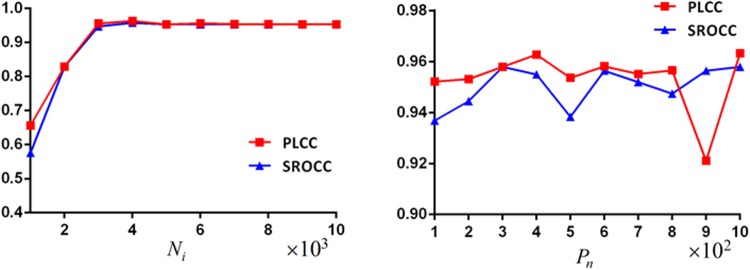
CNN prediction performance with *N*_*i*_ or *P*_*n*_ changes.

Fig 5 shows CNN performance when the iteration number (*N*_*i*_) varies from 10^3^ to 10^4^ and the patch number per image (*P*_*n*_) changes from 10^2^ to 10^3^. No much change is found after *N*_*i*_ reaches 4000. On the other side, *P*_*n*_ = 400 is a good point to tradeoff PLCC and SROCC. Therefore, we use *N*_*i*_ = 4000 and *P*_*n*_ = 400 hereafter.

Table 1 shows the CNN performance with regard to the kernel number (*K*_*n*_) and the kernel size ([*K*_*x*_, *K*_*y*_]). When *K*_*n*_ = 16, CNN performs well, while it is unstable when *K*_*n*_ = 32. On the other hand, prediction performance of CNN is insensitive to kernel size [*K*_*x*_, *K*_*y*_] changes. So we define *K*_*n*_ = 16 and *K*_*x*_ = *K*_*y*_ = 7.

**Table 1:** CNN performance with regard to kernel number and kernel size.

Kernel number	8	16	24	32
PLCC	0.9444	**0.9634**	0.9352	0.9298
SROCC	0.9519	**0.9543**	0.9504	0.9323
Kernel size	[3 3]	[5 5]	[7 7]	[9 9]
PLCC	0.9606	0.9508	**0.9632**	0.9319
SROCC	0.9669	**0.9684**	0.9579	0.9278

#### Parameters in GRNN and SVR

**Fig 6:**
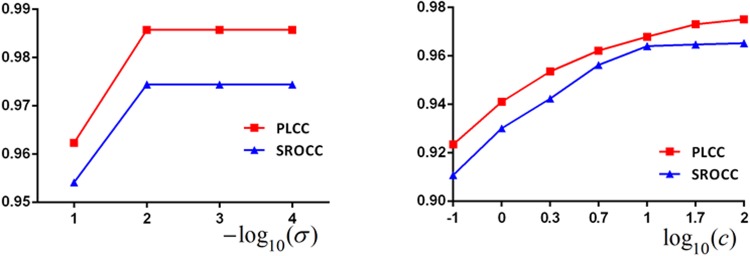
GRNN (left) and SVR (right) respectively perform when the spread parameter *σ* and the cost function *c* changes based on learned CNN features.

The spread parameter (*σ*) in GRNN and the cost function (*c*) in *ε*-SVR are studied with learned CNN features. Fig 6 shows PLCC and SROCC values when *σ* or *c* changes. The left plot indicates that when *σ* = 0.01, GRNN performs the best. The right shows that PLCC and SROCC increase when *log*_10_(*c*) increases, while when *log*_10_(*c*) > 1, SROCC keeps stable. Thus, *σ* = 0.01 in GRNN and *c* = 50 in *ε*-SVR.

### Learned CNN features

**Fig 7:**
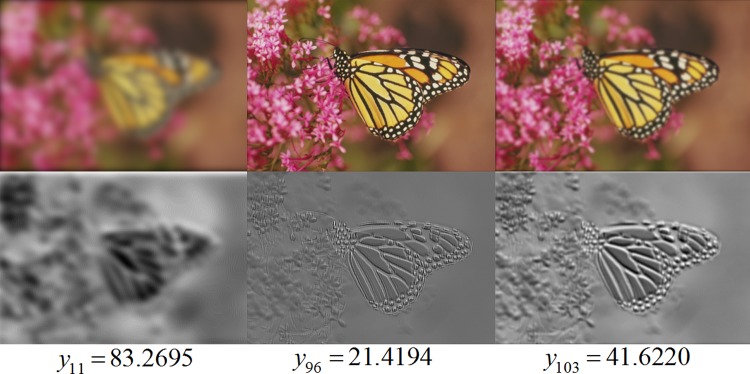
One trained kernel visualized by using “monarch.bmp”. After convolutional filtering with the trained kernel, edge structures is hard to notice in heavily blurred images (*y*_11_), while fine structures can be seen in relatively high-quality images (*y*_96_).

One trained kernel is visualized by using “monarch.bmp” in LIVE-II. Blurred images and their filtered results are shown in Fig 7. The top row shows Gaussian blurring images and the bottom row are images after convolutional filtering with the trained kernel. Underneath the filtered results are subjective scores, where lower scores indicate better visual quality. Compared to the relatively high-quality image (*y*_96_), fine structures vanish in low-quality images (*y*_11_ and *y*_103_).

### Algorithm performance

Table 2 summarizes the PLCC values and the highest values are marked in bold face. With handcrafted features, BIBLE [46] predicts the best, followed by SPARISH [44]. For CNNs, Kang’s CNN is instable. It achieves the best performance on TID2013 and the lowest value on CSIQ. For the proposed methods, CNN features with GRNN or SVR shows advantage. In general, retrieved features with SVR reaches an average PLCC value of 0.9435, and CNN features with GRNN gets 0.9377, followed by BIBLE (0.9251) and SPARISH (0.9217). Our CNN achieves an average PLCC of 0.9184.

**Table 2:** Performance evaluation with PLCC on Gaussian blurring images.

	LIVE-II	CSIQ	TID2008	TID2013	Overall
JNB [35]	0.8161	0.8061	0.6931	0.7115	0.7567
CPBD [36]	0.8955	0.8822	0.8236	0.8620	0.8658
S3 [38]	0.9434	0.9107	0.8542	0.8816	0.8975
FISH [39]	0.9043	0.9231	0.8079	0.8327	0.8670
LPC [40]	0.9181	0.9158	0.8573	0.8917	0.8957
SVC [42]	0.9416	0.9319	0.8556	0.8762	0.9013
MLV [43]	0.9429	0.9247	0.8583	0.8818	0.9019
SPARISH [44]	0.9595	0.9380	0.8891	0.9004	0.9217
ARISM [45]	0.9560	0.9410	0.8430	0.8954	0.9088
BIBLE [46]	0.9622	0.9403	0.8929	0.9051	0.9251
Kang’s CNN [55]	0.9625	0.7743	0.8803	**0.9308**	0.8875
Our CNN	0.9627	0.9255	0.8977	0.8875	0.9184
CNN features + GRNN	**0.9857**	**0.9473**	0.9059	0.9117	0.9377
CNN features + SVR	0.9730	0.9416	**0.9374**	0.9221	**0.9435**

Table 3 shows SROCC and bolded values indicate best predication monotonicity. BIBLE [46] shows superiority over algorithms based on handcrafted features, followed by SPARISH [44] and ARISM [45]. Kang’s CNN [55] achieves the highest SROCC on Gaussian blurring images from LIVE-II and TID2013, while it gets the second lowest SROCC on images from CSIQ among all metrics. On contrary, SROCC values from our CNN methods are robust on images from different databases. Particularly, CNN features with SVR outperforms other methods on CSIQ and TID2008. Furthermore, it ranks the second and the third place on TID2013 and LIVE-II, respectively. Generally, learned CNN features with SVR reaches an average SROCC of 0.9310, which is higher than CNN features with GRNN (0.9283), BIBLE (0.9160) and other methods.

**Table 3:** Performance evaluation of SROCC on Gaussian blurring images.

	LIVE-II	CSIQ	TID2008	TID2013	Overall
JNB [35]	0.7872	0.7624	0.6667	0.6902	0.7266
CPBD [36]	0.9182	0.8853	0.8414	0.8518	0.8742
S3 [38]	0.9436	0.9059	0.8480	0.8609	0.8896
FISH [39]	0.8808	0.8941	0.7828	0.8024	0.8400
LPC [40]	0.9389	0.9071	0.8561	0.8888	0.8977
SVC [42]	0.9343	0.9055	0.8362	0.8589	0.8837
MLV [43]	0.9312	0.9247	0.8548	0.8787	0.8974
SPARISH [44]	0.9593	0.9141	0.8869	0.8927	0.9133
ARISM [45]	0.9511	0.9261	0.8505	0.8982	0.9065
BIBLE [46]	0.9607	0.9132	0.8915	0.8988	0.9160
Kang’s CNN [55]	**0.9831**	0.7806	0.8496	**0.9215**	0.8837
Our CNN	0.9579	0.9048	0.8403	0.8376	0.8852
CNN features + GRNN	0.9744	0.9205	0.9163	0.9020	0.9283
CNN features + SVR	0.9646	**0.9253**	**0.9189**	0.9135	0.9310

### Time consumption

**Fig 8:**
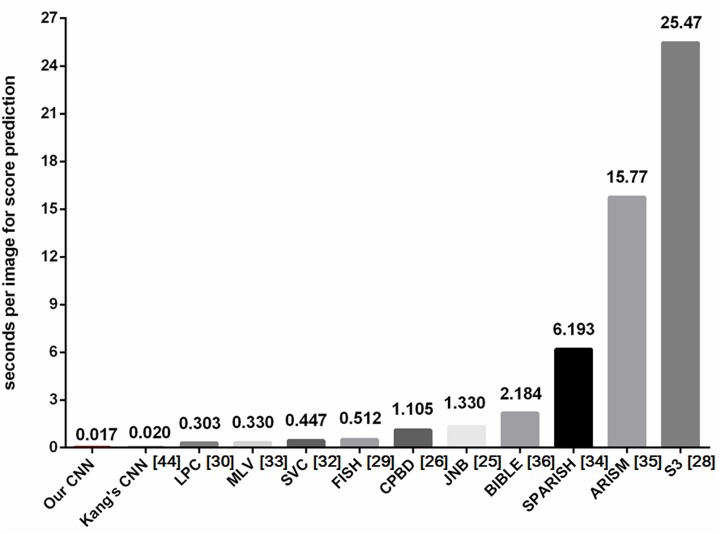
The time spent on score prediction of image sharpness. Several algorithms show promise in real-time image sharpness estimation.

The time spent on score prediction of image sharpness is shown in Fig 8. Among traditional methods, several algorithms show promise in real-time image sharpness estimation, such as LPC, MLV, SVC and FISH which require less than 1 *s*. For CNN-based methods, both models take about 0.02 *s* to rate an image. It should be noted that the major time of CNN models is spent on local contrast normalization which costs about 8 *s* for an image. Moreover, GRNN and SVR need time after the model is well trained. Fortunately, with the help of code optimization and advanced hardware, it is feasible to accelerate these algorithms and to satisfy real time requirement.

### Effect of color information

**Fig 9:**
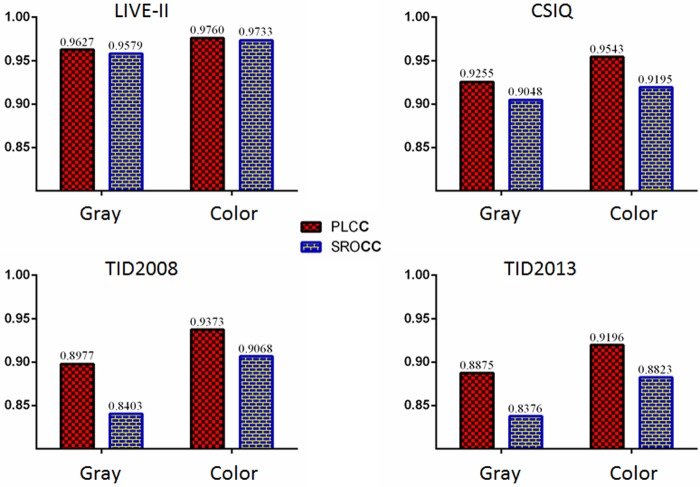
Effect of color information on our CNN. Compared to gray-scale input, color image input positively enhances our network’s prediction metrics.

Chroma is an important underlying property of human vision system [64, 65] and it is highly correlated with image quality perception [30, 44]. Effect of color information on image sharpness estimation is studied with our CNN. The performance of CNN with gray and color inputs is shown in Fig 9. It is observed that chromatic information positively enhances CNN’s performance on image sharpness estimation. The improved magnitude of PLCC ranges from 0.013 (LIVE-II) to 0.040 (TID2008). Meanwhile, the improved magnitude range of SROCC is from 0.014 (CSIQ) to 0.067 (TID2008).

### Future work

The proposed shallow CNN methods have achieved the state-of-the-art performance on simulated Gaussian blur images from four popular databases. Our future work will be to integrate handcrafted features and CNN features for improved prediction capacity. On the other hand, deeper networks will also be considered for representative features in image sharpness. In addition, with the public accessibility to the real-life blurring image databases of BID2011 [37] and CID2013 [66], it will be interesting to explore the proposed algorithm for more general and more practical applications [32, 67, 68].

## Conclusion

A shallow convolutional neural network is proposed to address blind image sharpness assessment. Its retrieved features with support vector regression achieves the best overall performance, indicating high correlation with subjective judgment. In addition, incorporating color information benefits image sharpness estimation with the shallow network.
